# Supplementary material for: Accelerating invasion potential of disease vector Aedes aegypti under climate change
Source: Nat Commun. 2020 May 1;11:2130. doi: 10.1038/s41467-020-16010-4 (PMC7195482; doi:10.1038/s41467-020-16010-4)
Supplement: Supplementary file 1 — Supplementary Information [file 41467_2020_16010_MOESM1_ESM.pdf]

SUPPLEMENTARY INFORMATION

The materials in this document supports results of "Accelerating invasion potential of disease vector *Aedes aegypti* under climate change" authored by Takuya Iwamura, Adriana Guzman-Holst and Kris A. Murray, published in Nature Communications in 2020.

Contents:

Supplementary Tables

Supplementary Table 1. Occurrence validation – Area Under the Curve (AUC)
Supplementary Table 2. Details of sites used for abundance validation
Supplementary Table 3. Comparison of global trends between a 5-yr and 10-yr averaging interval
Supplementary Table 4. Comparison between 200 mm/yr and 900 mm/yr precipitation threshold
Supplementary Table 5. Slope estimates for trends in LCC across comparison periods and latitudinal bands between 40N and 40S

Supplementary Figures

Supplementary Figure 1. Abundance validation results
Supplementary Figure 2. Regions for computing life-cycle completion (LCC) trends
Supplementary Figure 3. Trends in LCC across comparison periods and latitudinal bands between 40N and 40S
Supplementary Figure 4. Slope estimates (rate of change, in units of LCC per month, reflecting underlying monthly time series data) for trends in LCC across comparison periods and latitudinal bands between 40N and 40S
Supplementary Figure 5. Description of phenology model for *Ae. aegypti*
Supplementary Figure 6. Sensitivity analysis on the model parameters
Supplementary Figure 7. The global averages based on GDDP-NEX and ERA5 between 1980-2005
Supplementary Figure 8. Spatial distribution of LCC with NEX-GDDP vs ERA5 (1980-2005)

Supplementary Table 1. Occurrence validation – Area Under the Curve (AUC). The model output (LCC) was evaluated against occurrence records of *Ae. aegypti* (Kraemer *et al.* 2015). The predicted LCC results averaged over the years 2001-2010 were compared to known observation records of *Ae. aegypti* restricted to the same period and summary statistics computed. The Area Under the Curve (AUC) or the Receiver Operating Characteristic (ROC) was examined for the model's ability to discriminate areas of occurrences from areas where it has not been observed, as represented by randomly generated pseudo-absence points. For country-level validation, we did not use Kraemer et al., background dataset which contains the observations on the presence of other mosquito species because many of the countries with high *Ae. aegypti* observations often lack observations for other species, leading to AUC inflation. To calculate the AUC, the life-cycle completion values were first standardised on a 0 – 1 scale, where 1 equates to the maximum number of life cycles. We could not use Kappa as in the global analysis, since it requires a threshold to be set for discriminating presence from absence and the idiosyncrasies at country level means such a threshold would vary. We only examined countries with more than 150 data points.

Country	AUC
Taiwan	0.99
Thailand	0.84
Mexico	0.82
Australia	0.80
U.S.A.	0.79
Argentina	0.77
Vietnam	0.74
Malaysia	0.63
India	0.55
Indonesia	0.49
Cuba	0.47
Brazil	0.35

Supplementary Table 2. Details of sites used for abundance validation. (Lozano-Fuentes et al., 2012) and Moreno-Madriñán et al., 2014). The study was conducted in 2011 and represents the best available case study that we are aware of focusing on *Ae. aegypti* abundance. The surveys spanned a relatively large geographic area (300x100km) and, critically, employed the same sampling methodology across all surveys. We utilised data from 10 villages for analysis following Moreno-Madriñán et al. (2014).

Sites	Latitude	Longitude	Elevation (m)	Observations
Veracruz City	19.181°	-96.163°	18	792
Cordoba	18.894°	-96.953°	860	570
Coatepec	19.454°	-96.959°	1203	602
Ciudad Mendoza	18.806°	-97.187°	1338	350
Xalapa	19.545°	-96.912°	1419	149
Acultzingo	18.718°	-97.304°	1695	212
Maltrata	18.808°	-97.277°	1714	7
Perote	19.564°	-97.248°	2417	0
Puebla City	19.038°	-98.203°	2141	3
Atlixco	18.911°	-98.432°	1831	164

Supplementary Table 3. Comparison of global trends between a 5-yr and 10-yr averaging interval. The global trends of LCC between 1950s – 2000s as well as 2000s – 2050s are shown below. We confirmed that the choice of time window used for averaging (5-yr and 10-yr periods) does not appreciably affect our interpretation of the trends, with an average 1.9% difference between the two interval windows.

	5-yr window	10-yr window
1950s – 2000s increase rate*	7.0%	9.0%
2000s – 2050s increase rate (RCP4.5)	17.1%	16.9%
2000s – 2050s increase rate (RCP8.5)	24.3%	24.5%
1950s – 2050s increase rate (RCP4.5)	26.0%	27.4%
1950s – 2050s increase rate (RCP8.5)	33.8%	40.1%

Supplementary Table 4. Comparison between 200 mm/yr and 900 mm/yr precipitation threshold*. We compared different precipitation requirements for the life cycle completion for *Ae. aegypti*. The results presented in the main text are based on 200 mm/yr threshold, which corresponds to the precipitation level below which approximately 1% of the historical observations occur (Kraemer et al. 2015). Here, we used 900 mm per year threshold, below which 5.3% of historical observations occur, to calculate LCC to establish whether choice in the precipitation threshold affected the interpretation of our key results. When averaged globally, both thresholds yielded similar increases in LCC during the period 1950s – 2000s as well as for the projections (2000s – 2050s) for each RCP emissions scenario (Supplementary Table 3). Note that LCC values themselves were generally lower at the 900 mm threshold compared with the 200 mm (as much as 36% lower), likely because the areas with lower precipitation tend to have higher temperatures.

	200 mm threshold	900 mm threshold
1950s – 2000s increase rate (%)	8.5%	7.9%
2000s – 2050s increase rate (%), RCP4.5	15.3%	14.9%
2000s – 2050s increase rate (%), RCP8.5	21.1%	21.5%

* Comparison is made using 1 GCM (bcc-csm1-1) for both 200 mm and 900 mm scenarios in order to avoid confounding effect of averaging different GCMs.

Supplementary Table 5. Slope estimates for trends in LCC across comparison periods and latitudinal bands between 40N and 40S. Seasonal Kendall trend tests and Sen slope estimation was conducted with the *EnvStats* package in R (see Supplementary Figure 4). All trends assessed except one (20-30N 1950-2000, p=0.127) were positive and significant at p<0.001, as indicated by CIs not including 0 (two-tailed test). For each test, *n*=600 (50 years x 12 months). Trend slopes were smallest in the historical data and at higher and lower latitudes (see Supplementary Figure 4). Slopes were steeper in the future projections, with higher emissions, and in the central (subtropical and tropical) bands.

Latitude	Series	tau	slope	p-value
30-40N	1950-2000	0.1392	0.0001	< 0.0001
20-30N	1950-2000	0.0428	0.0001	0.1273
10-20N	1950-2000	0.2070	0.0006	< 0.0001
0-10N	1950-2000	0.3285	0.0015	< 0.0001
30-40S	1950-2000	0.2157	0.0002	< 0.0001
20-30S	1950-2000	0.2853	0.0008	< 0.0001
10-20S	1950-2000	0.3543	0.0011	< 0.0001
0-10S	1950-2000	0.3720	0.0014	< 0.0001
30-40N	2000-2050 RCP4.5	0.5467	0.0013	< 0.0001
20-30N	2000-2050 RCP4.5	0.6030	0.0013	< 0.0001
10-20N	2000-2050 RCP4.5	0.6511	0.0022	< 0.0001
0-10N	2000-2050 RCP4.5	0.6564	0.0035	< 0.0001
30-40S	2000-2050 RCP4.5	0.4741	0.0007	< 0.0001
20-30S	2000-2050 RCP4.5	0.4303	0.0017	< 0.0001
10-20S	2000-2050 RCP4.5	0.6562	0.0027	< 0.0001
0-10S	2000-2050 RCP4.5	0.7648	0.0036	< 0.0001
30-40N	2000-2050 RCP8.5	0.6405	0.0019	< 0.0001
20-30N	2000-2050 RCP8.5	0.7283	0.0021	< 0.0001
10-20N	2000-2050 RCP8.5	0.7497	0.0034	< 0.0001
0-10N	2000-2050 RCP8.5	0.6963	0.0047	< 0.0001
30-40S	2000-2050 RCP8.5	0.5335	0.0009	< 0.0001
20-30S	2000-2050 RCP8.5	0.4415	0.0016	< 0.0001
10-20S	2000-2050 RCP8.5	0.7004	0.0035	< 0.0001
0-10S	2000-2050 RCP8.5	0.8077	0.0054	< 0.0001

Supplementary Figure 1. Abundance validation results. Correlation between abundance estimates from Supplementary Table 1 and model predictions of LCC for the same locations (Pearson's r = 0.752, r^2 = 0.571, p = 0.011). Abundance of each site was collected during the summer months of 2011 (Lozano-Fuentes et al., 2012). LCC values were calculated from a 2011 climatic dataset, and averaged over the 4 GCMs under RCP4.5.

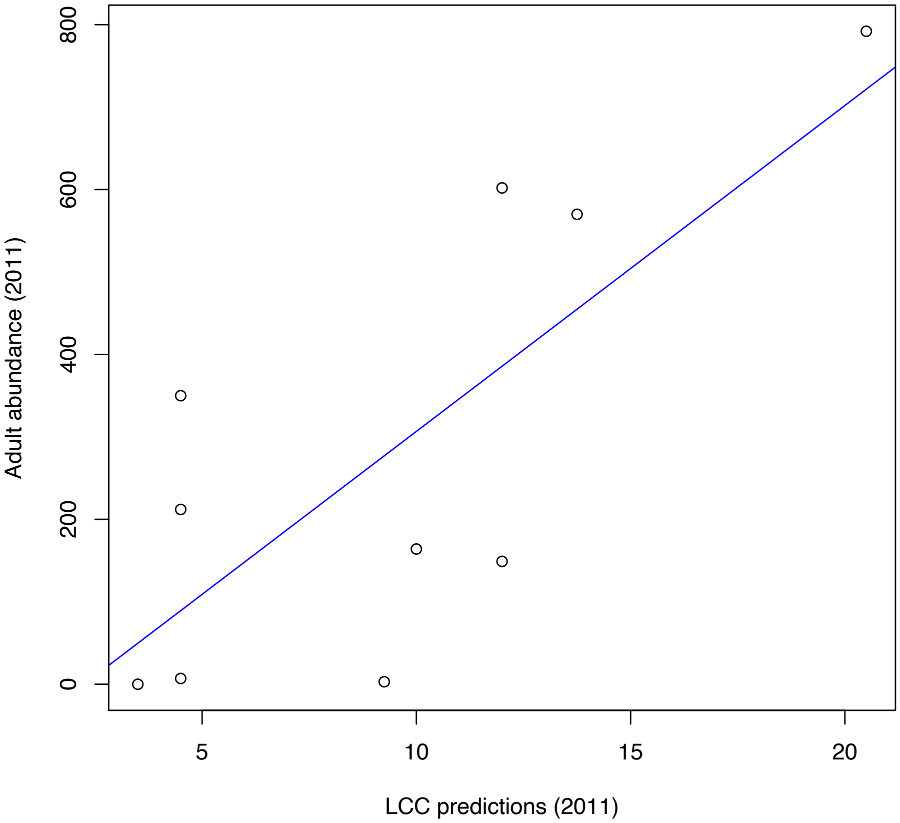

Supplementary Figure 2. Regions for computing life-cycle completion (LCC) trends. a) Continental - we divided the world terrestrial areas into 13 regions, following the United Nation's continental categories. We combined Eastern and Southern Africa, and Western and Central Africa to increase area size and reduce the number of regions. Russia is grouped with Central Asia. b) Climatic – the world is divided into 5 climatic types, following level 1 classifications of the Köppen climate classification (Kottek et al. 2006).

a)

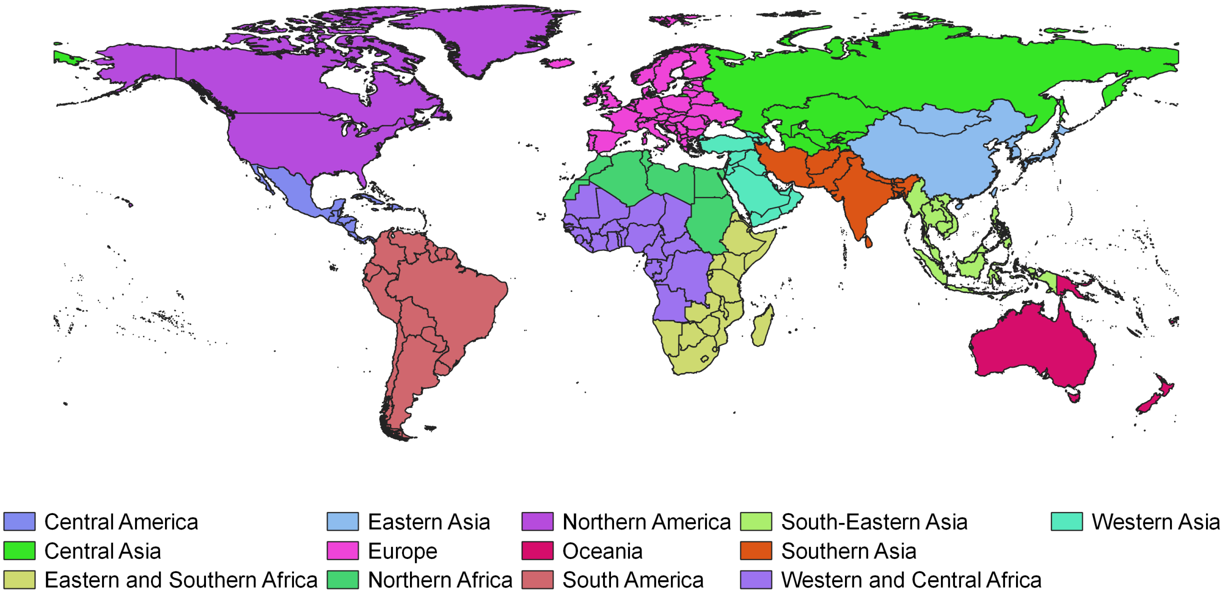

b)

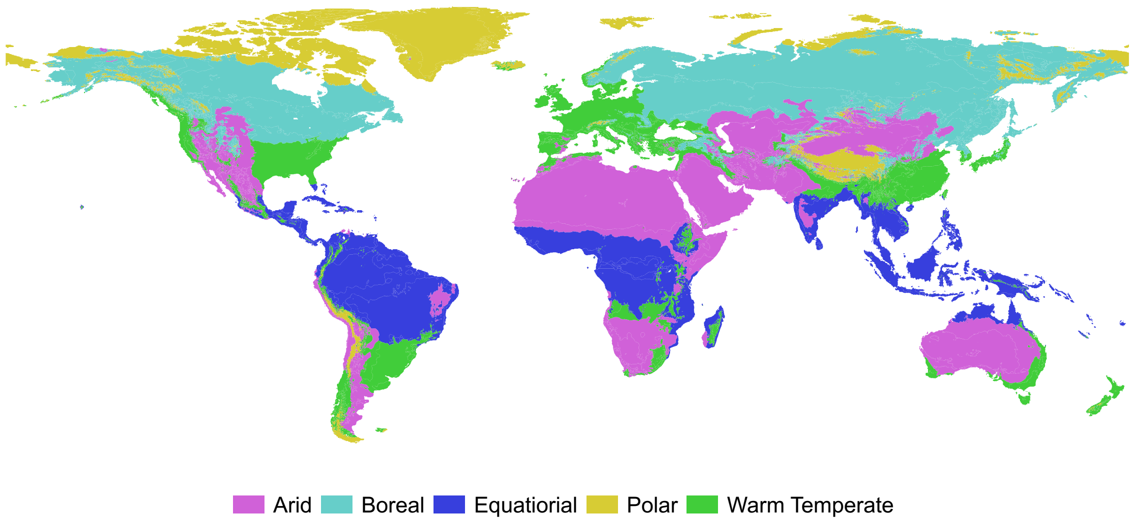

Supplementary Figure 3. Trends in LCC across comparison periods and latitudinal bands between 40N and 40S. Solid trend lines are loess smoothers on the underlying trend data. Underlying trend data were calculated by decomposing raw monthly (seasonal) time series data into its constituent components (trend, seasonality, random) using the *ggseas* package in R. The historical trend is calculated on data from 1950-2000. Projected trends are calculated on data from 2000-2050 for each RCP scenario. Trend slope statistics are presented in Supplementary Figure 4.

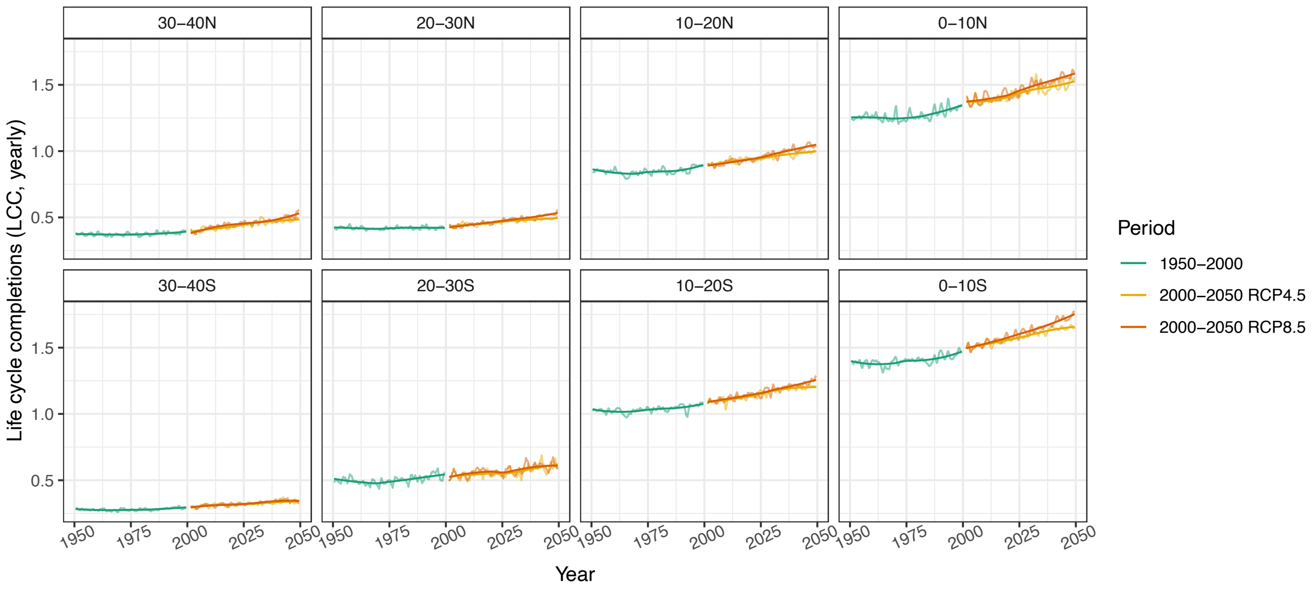

Supplementary Figure 4. Slope estimates (rate of change, in units of LCC per month, reflecting underlying monthly time series data) for trends in LCC across comparison periods and latitudinal bands between 40N and 40S. Seasonal Kendall trend tests and Sen slope estimation was conducted with the *EnvStats* package in R. All trends assessed except one (20-30N 1950-2000, p=0.127) were positive and significant at p<0.001, as indicated by CIs not including 0 (two-tailed test) (see Supplementary Table 5). For each test, *n*=600 (50 years x 12 months). Trend slopes were smallest in the historical data and at higher and lower latitudes. Slopes were steeper in the future projections, with higher emissions, and in the central (subtropical and tropical) bands.

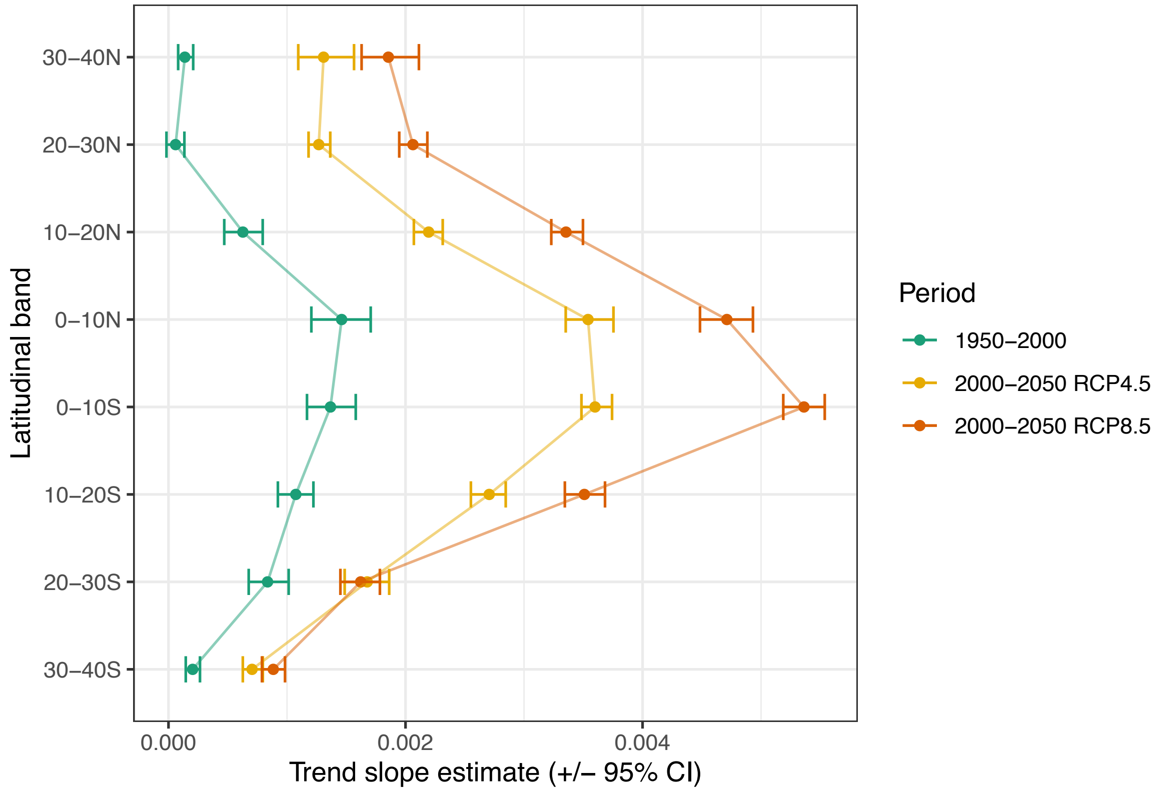

Supplementary Figure 5. Description of phenology model for *Ae. aegypti.* The phenology model was designed following this diagram and implemented as computer code in *R*. Rectangles represent different development stages of *Aedes* mosquitoes, and diamonds indicate the specific conditions for *Ae. aegypti.* See main text for further details.

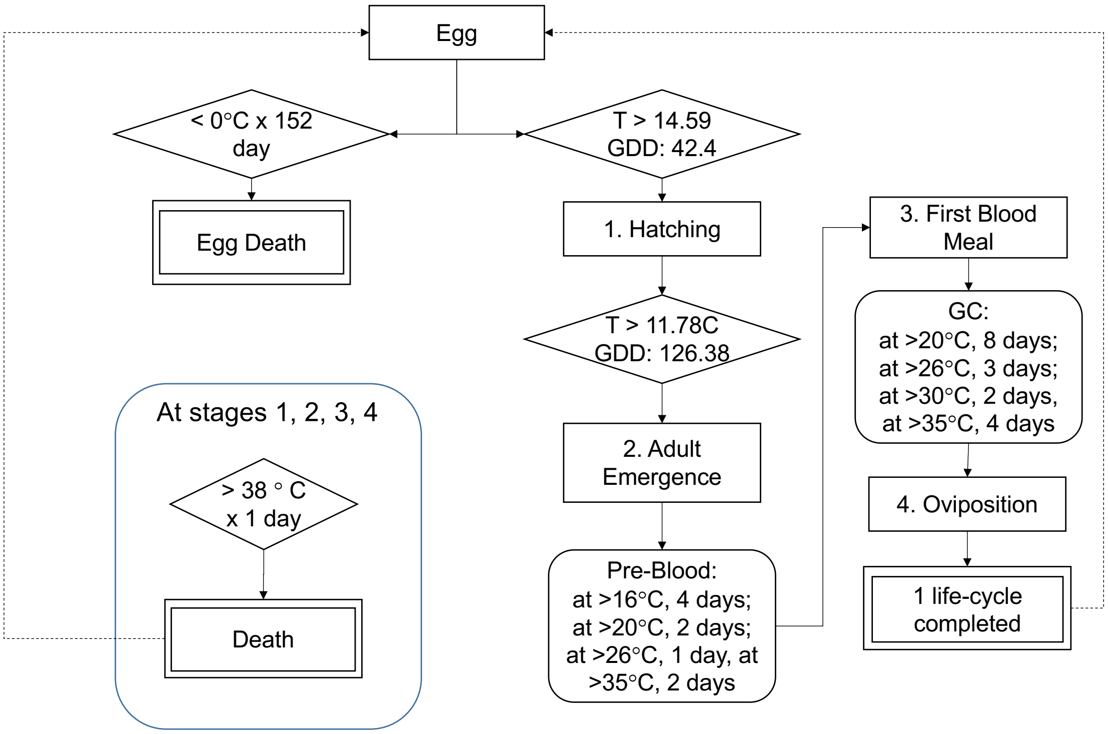

Supplementary Figure 6. Sensitivity analysis on the model parameters.

a. Oviposition (incl. blood feeding). Default setting for oviposition (including blood feeding) is conditioned as non-linear curve against temperature based on literature (see Methods). Here, we compared models in which this value was decreased or increased (75%, 125% and 150%) to explore the impacts of oviposition period on predicted LCC. The results indicate that while the value affects LCC by adding (or subtracting) days to complete life cycles, the overall trends remained the same.

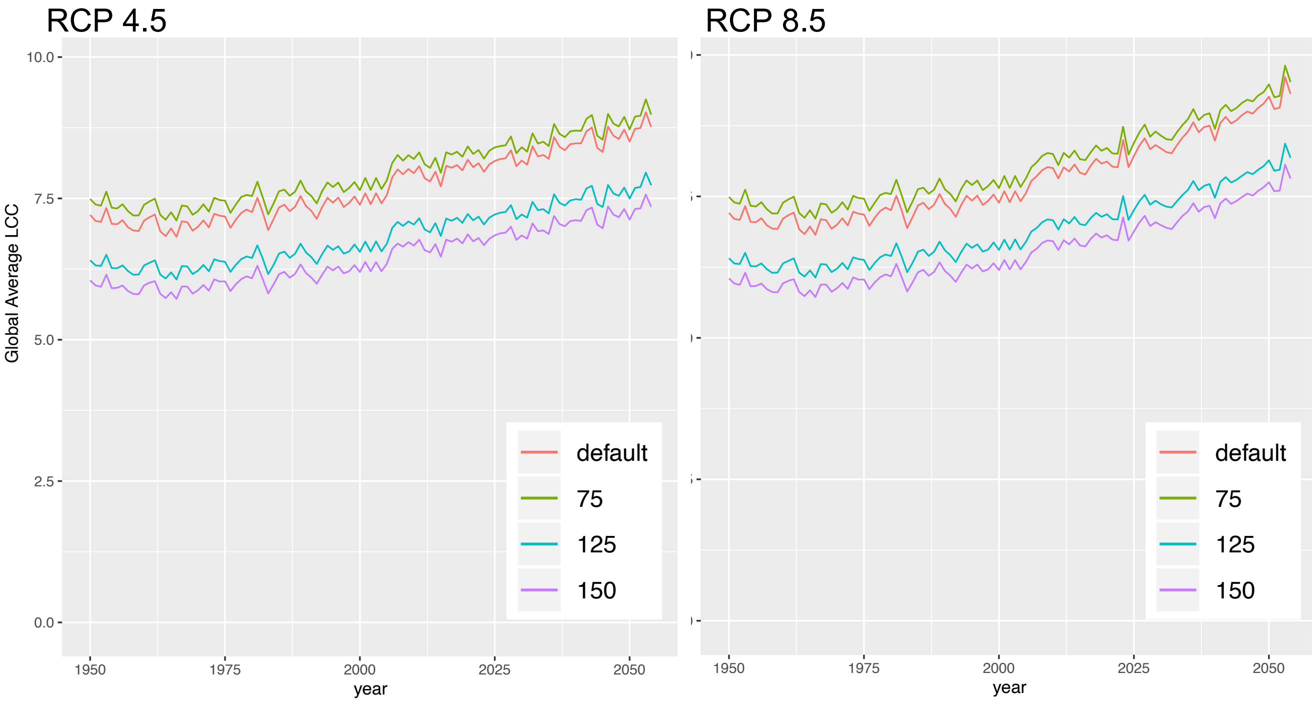

b. Coldkill. Coldkill occurs when the temperature falls below a certain threshold. At this temperature, both hatched larvae and adults die while eggs will survive. If the same conditions last more than 150 days, it is assumed that the eggs cannot survive and thus mosquitoes cannot colonize. As a default, we set the threshold for coldkill as 0°C based on the literature (see Methods). Here we compared the results at -5°C, +5°C and +10°C. There was little effect on model outputs of varying the coldkill threshold.

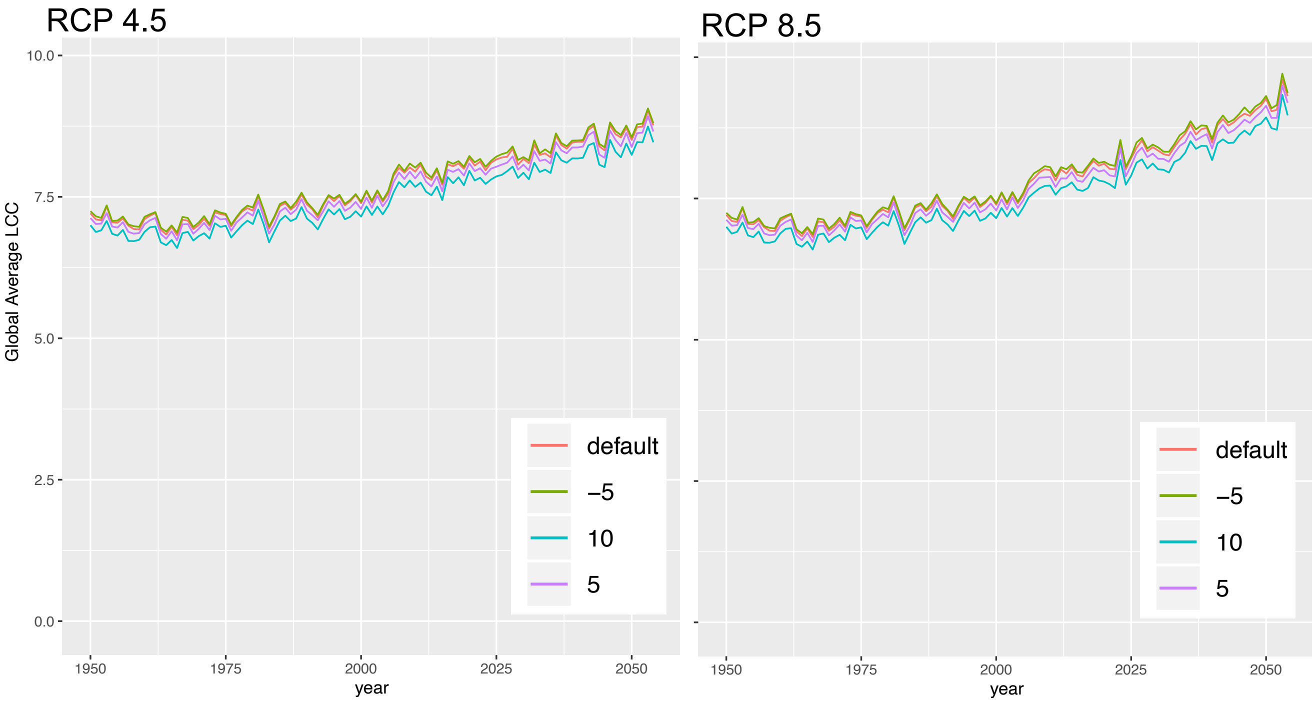

c. **Heatkill.** Heatkill occurs when the temperature exceeds a certain threshold. At this temperature, the adult will not die but cannot reproduce. As a default, we set the threshold for heatkill as 38°C based on the literature (see Methods). We conducted sensitivity analysis from the default to explore the effect of varying this threshold (36°C and 40°C). There was little effect on model outputs of varying the heatkill threshold.

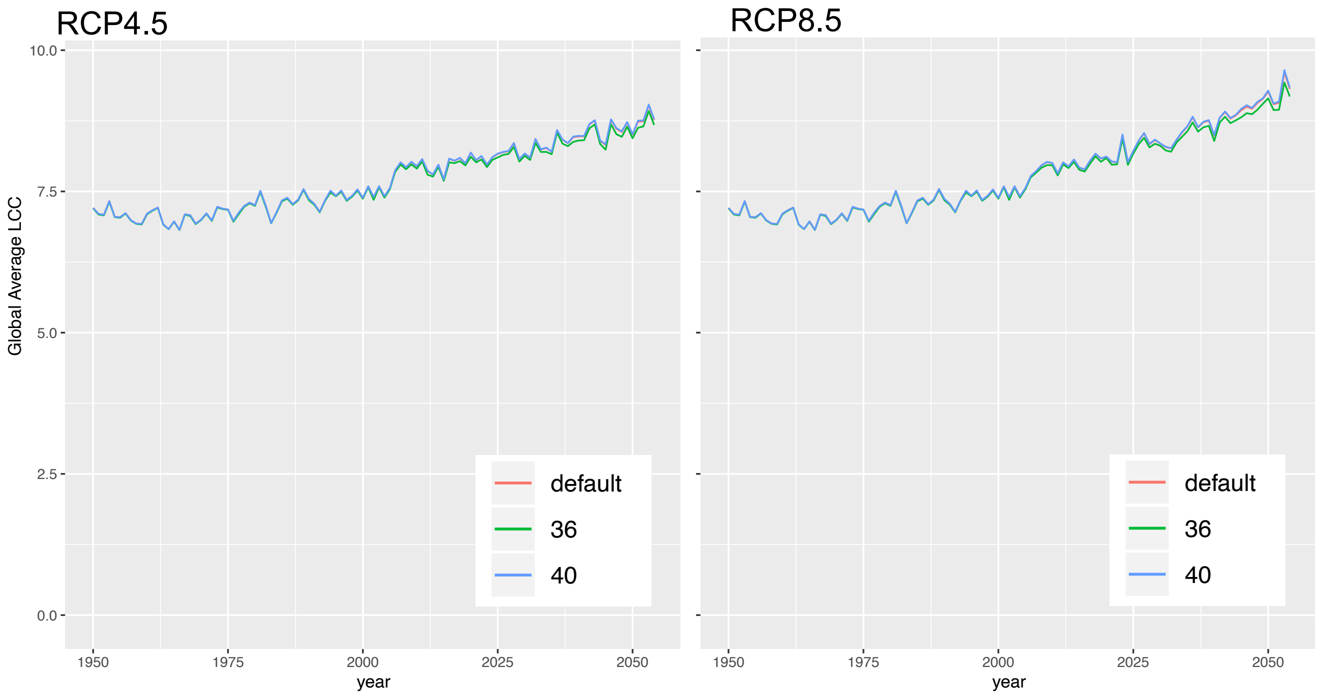

Supplementary Figure 7. The global averages based on GDDP-NEX and ERA5 between 1980-2005. The ERA5 dataset is based on observation records and provides high quality historical data for the period 1979 to 2019. ERA5 is available hourly at 30 by 30 km globally. Here we compared our results based on the NASA GDDP-NEX dataset (main text) with results from ERA5 between 1980 and 2005 in order to test for an effect of the underlying climate data set on the results. To facilitate handling of the large datasets in ERA5, we selected data at 15:00hrs to represent the daily Tmax and 06:00hrs to represent the daily Tmin to correspond with the equivalent measures provided in the NASA NEX dataset. While the outputs are not equivalent as expected (GDDP is based on GCMs, and ERA 5 is calculated from observation), they were strongly correlated between datasets ($r^2 = 0.53$, $p < 0.001$). The differences in LCC are on average 3.2% lower for ERA5 than GDDP during the period examined. The effect of this difference was most noticeable in America and some of the tropical regions, where ERA5 produces more nuanced temperature gradients (see Supplementary Figure 8).

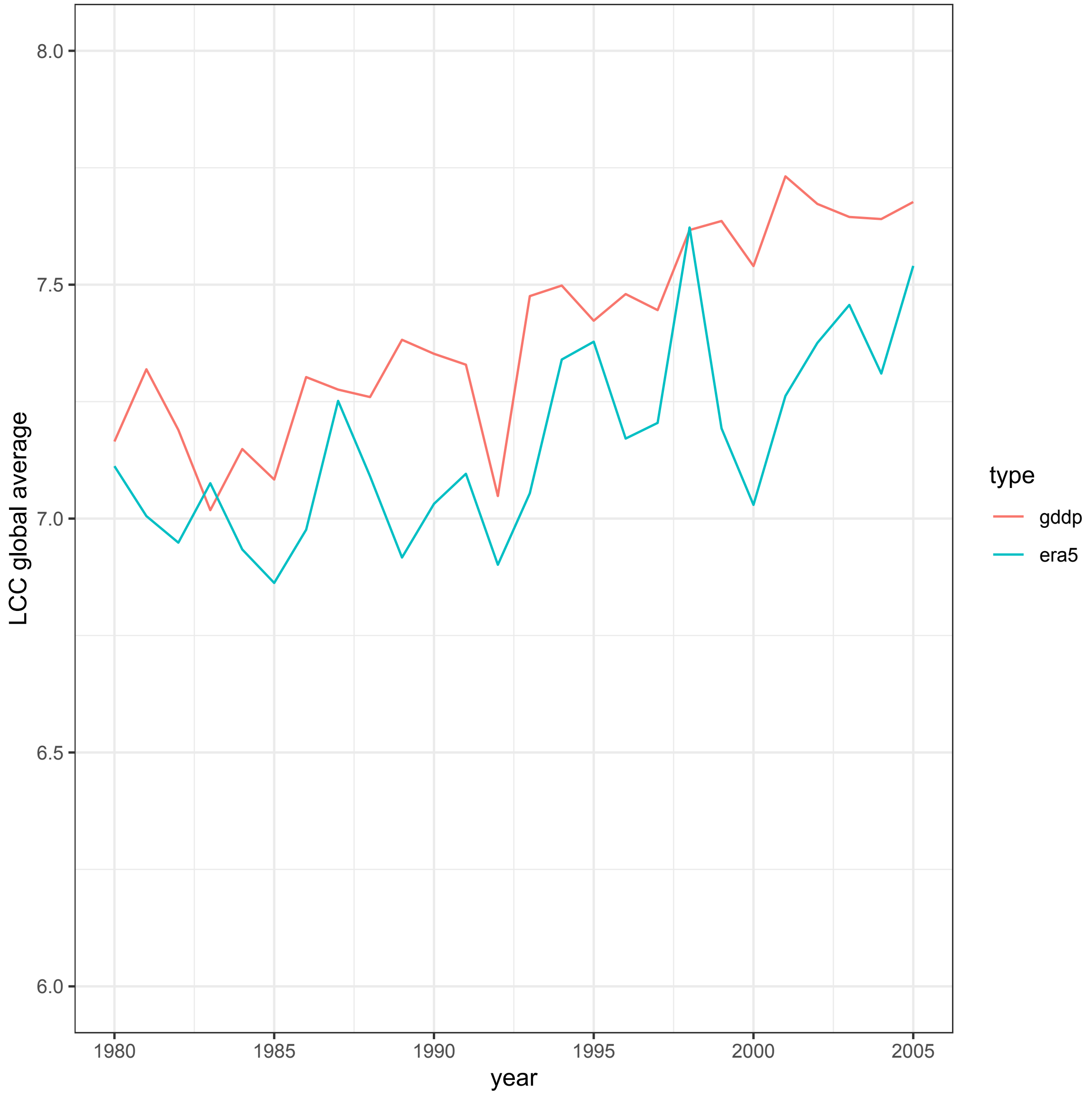

Supplementary Figure 8. Spatial distribution of LCC with NEX-GDDP vs ERA5 (1980-2005). The global distribution of mean LCC (averaged over the period1980-2005) using a) NEX-GDDP and b) ERA5 datasets. c) difference between the two, calculated by (GDDP-ERA5)/ERA5. Overall patterns at the global scale are similar (a and b). The distribution of the differences between NEX-GDDP and ERA 5 reveal that GDDP tends to yield higher average LCC in tropical regions (e.g. Amazon and Congo basins). ERA 5 has higher LCC in dry and cold regions (e.g. Australia, Middle East).

a)

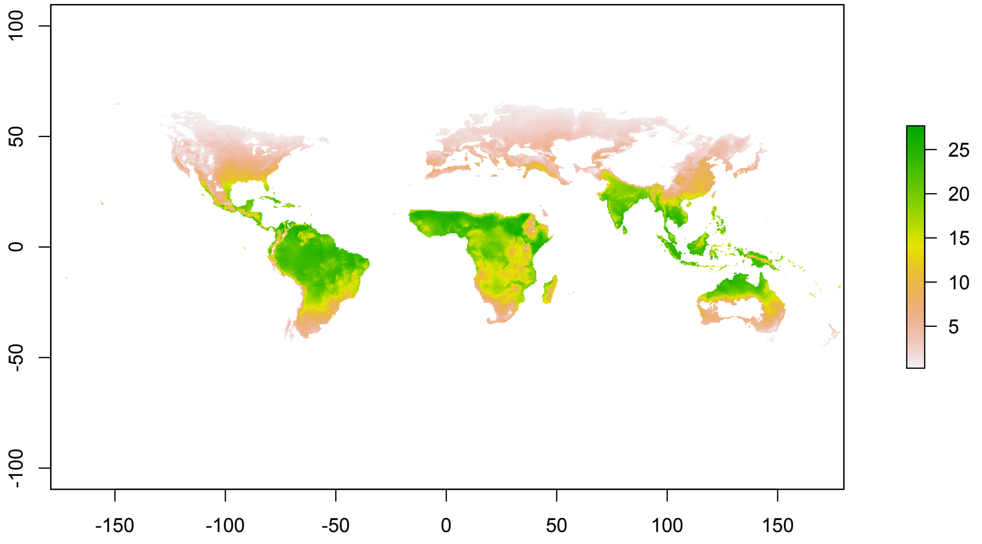

b)

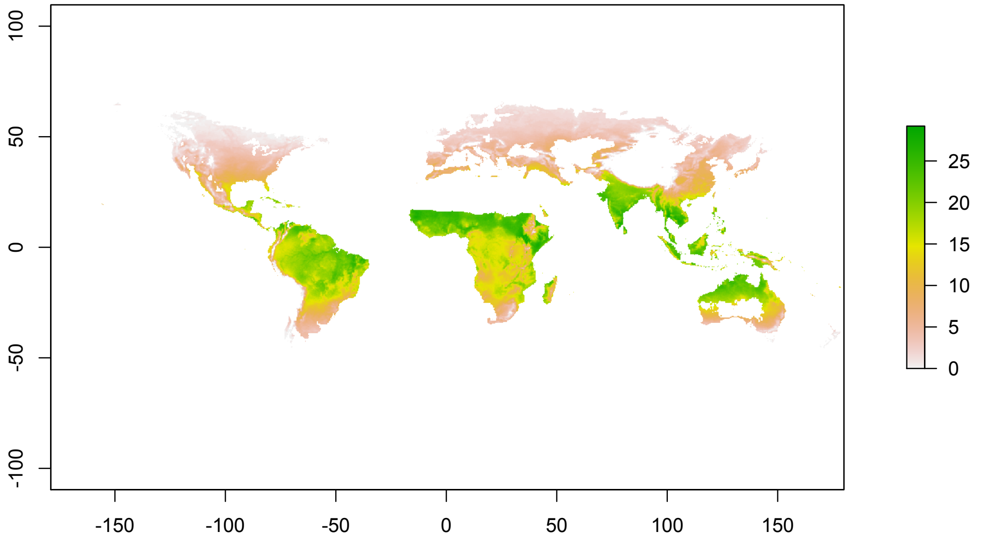

c)

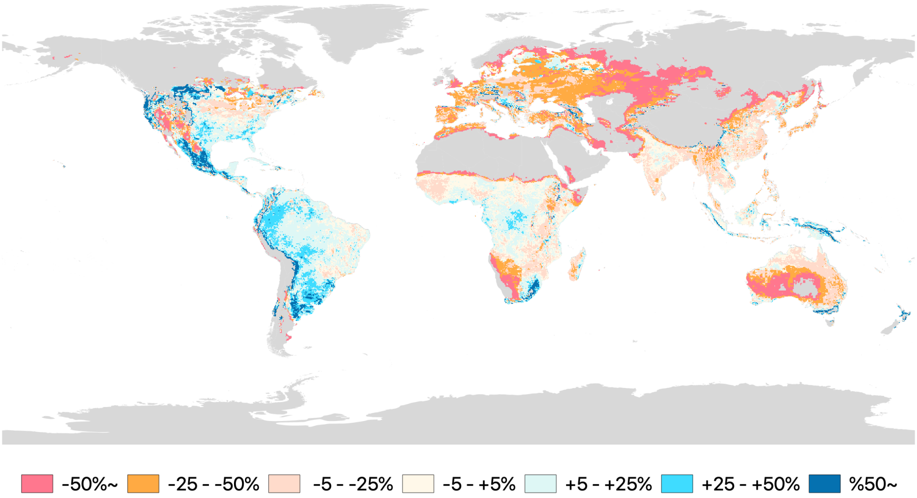

Supplementary Information References

Kraemer, M. U. G. *et al.* The global distribution of the arbovirus vectors Aedes aegypti and Ae. albopictus. *Elife* (2015). doi:10.7554/elife.08347

Lozano-Fuentes, S. *et al.* The dengue virus mosquito vector *Aedes aegypti* at high elevation in México. *Am. J. Trop. Med. Hyg.* (2012). doi:10.4269/ajtmh.2012.12-0244

Moreno-Madriñán, M. *et al.* Correlating remote sensing data with the abundance of pupae of the dengue virus mosquito vector, *Aedes aegypti*, in central Mexico. *ISPRS Int. J. Geo-Information* (2014). doi:10.3390/ijgi3020732

Kottek, M. *et al.* World Map of the Koppen-Geiger climate classification updated. *Meteorol. Zeitschrift* (2006). doi: 10.1127/0941-2948/2006/0130
